# Deciphering the Causes of IbfA-Mediated Abortive Infection in the P22-like Phage UAB_Phi20

**DOI:** 10.3390/ijms26104918

**Published:** 2025-05-20

**Authors:** Júlia López-Pérez, Pilar Cortés, Susana Campoy, Ivan Erill, Montserrat Llagostera

**Affiliations:** 1 Molecular Microbiology Group, Departament de Genètica i de Microbiologia, Universitat Autònoma de Barcelona, 08193 Cerdanyola del Vallès, Barcelona, Spain; julia.tickles@gmail.com (J.L.-P.); susana.campoy@uab.cat (S.C.); montserrat.llagostera@uab.cat (M.L.); 2 Departament d’Enginyeria de la Informació i de les Comunicacions Àrea de Ciències de la Computació i Intel·ligència Artificial, Universitat Autònoma de Barcelona, 08193 Cerdanyola del Vallès, Barcelona, Spain; ivan.erill@uab.cat

**Keywords:** *Salmonella*, phage therapy, bacterial defense mechanism, IncI1α conjugative plasmid, *ibfA*, abortive infection

## Abstract

The study of bacterial defense mechanisms against phages is becoming increasingly relevant due to their impact on the effectiveness of phage therapy. Employing a multifaceted approach that combines bioinformatics, molecular microbiology, TEM microscopy, and conventional microbiology techniques, here, we identify the *ibfA* gene as a novel defense factor targeting the virulent phage UAB_Phi20, acquired by *Salmonella* Typhimurium through lateral transfer on the IncI1α conjugative plasmid pUA1135 after oral phage therapy in broilers. IbfA, a two-domain protein containing ATPase and TOPRIM domains, significantly reduces UAB_Phi20 productivity, as indicated by decreased EOP, ECOI, and a diminished burst size, potentially reducing cellular viability without causing observable lysis. Our results indicate that IbfA enhances the transcription of early genes, including the antirepressor *ant*, which inhibits the C2 repressor of the lytic cycle. This may cause an imbalance in Cro/C2 concentration, leading to the observed reduction in the transcription of late genes encoding structural and cellular lysis proteins, and resulting in the abortion of UAB_Phi20 infection.

## 1. Introduction

Phages are viruses that infect bacteria, and they are genetically the most diverse biological entities on Earth [1], greatly outnumbering their hosts in nature [2]. Phage therapy offers a convincing strategy to combat the increasing crisis of multidrug-resistant bacterial infections. Bacteriophages are viruses with many advantages that make them a highly capable solution for the treatment of these infections, such as their ability to self-replicate inside their hosts and their ability to selectively infect and lyse specific bacterial species, without affecting the beneficial commensal microbiota within the host, a significant advantage over broad-spectrum antibiotics. This approach provides a highly targeted means of eliminating pathogens that have developed resistance to conventional antibiotics. As research and clinical applications advance, phage therapy stands poised to become an increasingly vital component of our antimicrobial arsenal.

However, the evolutionary arms race between phages and bacteria has resulted in the development of a wide range of bacterial strategies to combat phage infection, posing an important challenge for the extensive application of phage therapy in human and animal health. The study of these defense strategies is crucial for the safe use of phages in phage therapy.

The number of reported phage defense strategies has grown exponentially in the last few years. These strategies are very diverse, and they disrupt the phage life cycle at different stages [3,4]. The first line of defense against phages is the prevention of adsorption to their receptors, either through mutation or deletion of the receptor genes, masking the receptor with extracellular polymeric substances or using outer membrane vesicles as a decoy [5]. DNA injection is prevented by mechanisms such as superinfection exclusion (Sie) [6]. Once phage DNA has been injected, restriction-modification (RM) [7], phage growth limitation (PgI) [8], bacteriophage exclusion (BREX) [9], defense island associated with RM (DISARM) [10], prokaryotic argonautes (pAgos) [11], restriction by an adenosine deaminase acting on RNA (RADAR) [12,13] or clustered regularly interspaced short palindromic repeats and associated proteins (CRISPR-Cas) [14] act through different mechanisms to inhibit phage genome replication. The transcription of phage genes can also be the target of bacterial defenses. For example, prokaryotic viperins (pVips) [15] produce several modified ribonucleotides that inhibit phage transcription by acting as RNA chain terminators. On the other hand, abortive infection (Abi) systems cause cell death before phages have completed their life cycle, avoiding the spread of the infection in the population. These systems typically target replication, transcription, or translation, and they are mechanistically diverse. One of the classical Abi mechanisms are toxin-antitoxin (TA) systems [16,17]. More recently described Abi systems include cyclic-oligonucleotide-based antiphage signaling systems (CBASS) [18], Thoeris [19,20], bacterial gasdermins [21], and retrons [22]. In addition to these well-characterized phage defense systems, there is a growing list of systems with unknown mechanisms that are known to interfere with the phage cycle [19,23].

In previous work by our group [24], we used bacteriophage UAB_Phi20 (a P22-like bacteriophage) in combination with two other phages (UAB_Phi78 and UAB_Phi87) in a phage therapy study performed on broiler chickens experimentally contaminated with *Salmonella enterica* serovar Typhimurium ATCC14028 resistant to rifampicin (Rif^R^). This study achieved a reduction in *Salmonella* concentration in the ceca of broilers of 2.8 log_10_ at 6 days after a daily phage treatment of the animals. In the study, several *Salmonella* variants with reduced phage susceptibility were isolated from the broilers’ ceca. Interference with the phage multiplicative cycle in these variants was found to be associated with IncI1α large conjugative plasmids, acquired by lateral transfer from the broilers’ gut microbiota [25]. One of the variants, named IC5, harbored one large conjugative plasmid pUA1135 (96,685 bp) and exhibited reduced susceptibility to phage UAB_Phi20 and to the other two phages of the cocktail. *Salmonella* phage UAB_Phi20 is a virulent phage belonging to the *Lederbergvirus* genus. It has an icosahedral head of about 60 nm and a noncontractile short tail (13 ± 0.7 nm) [26]. Its genome is a linear double-stranded DNA (dsDNA) molecule of 41,809 base pairs (bp) in length with circularly permuted direct terminal repeats [27]. The UAB_Phi20 genome is highly similar to *Salmonella* phage P22 [27], and although it encodes all the components necessary for a lysogenic cycle, it was shown to be a virulent phage [27].

In this work we identify the *ibfA* gene encoded in plasmid pUA1135 as the agent responsible for interfering with the UAB_Phi20 phage. This gene is homologous to the *ibfA* gene found in plasmid R64, which has been related to changes in phage type [28] and interference with coliphages PhiI and λ, and *S*. Typhimurium phages P22 and St [29,30]. However, the mechanism of action of this gene has not been elucidated. Here, we show that IbfA reduces the efficiency of plating (EOP), the efficiency of center of infection (ECOI), and the burst size of UAB_Phi20, leading to decreased cellular viability without detectable cell lysis. Our findings suggest that IbfA, either directly or indirectly, boosts the transcription of early genes such as the antirepressor *ant*, which inhibits the C2 repressor of the lytic cycle. This disruption likely disturbs the Cro/C2 balance, leading to a decrease in transcription of late genes involved in phage assembly and cellular lysis proteins, and ultimately causing the abortion of UAB_Phi20 infection.

## 2. Results

### 2.1. Unravelling the Plasmid Region That Disrupts the UAB_Phi20 Multiplicative Cycle

**Figure 1:**
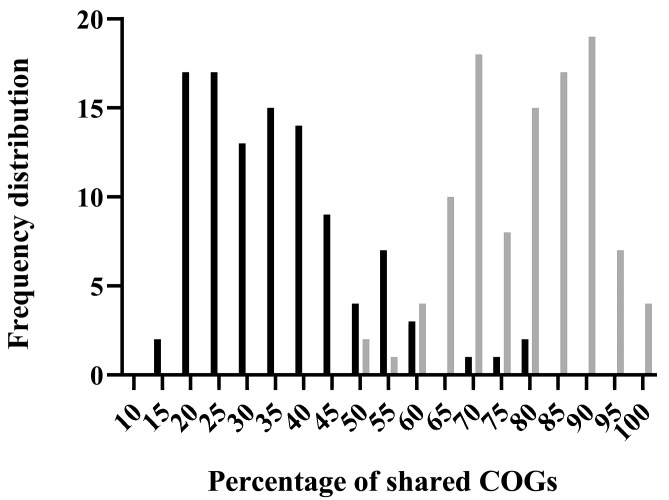
Frequency distribution of the fraction of shared COGs in pairwise comparisons of the variable region (black) and the rest of pUA1135 plasmid sequence (grey) with a set of 30 pUA1135 homologs.

Phage defense systems are known to often localize to the high-variability regions of plasmid and chromosome sequences [31,32,33]. A tBLASTN search against the PLSDB database revealed that the pUA1135 plasmid (NCBI accession number: MW590592.1) of *S*. Typhimurium IC5 strain [25] contains a region, between nucleotides 2000 (*repA* gene) and 18,500 (*parA* gene), that shows considerably more variation than the rest of the plasmid sequence. Homologs of pUA1135 exhibited numerous rearrangements, insertions, and deletions within this region. To quantify this variability, we performed a study of the frequency of distribution of shared COGs through pairwise comparisons of the variable region and the rest of the plasmid (Figure 1). The results demonstrated that the percentage of shared COGs is much lower when comparing variable regions between plasmids than when comparing non-variable regions. A COG inventory of the variable region of these homologous plasmids revealed a preponderance of transposases, further underscoring the inherent variability in this region (Table S1).

**Figure 2:**
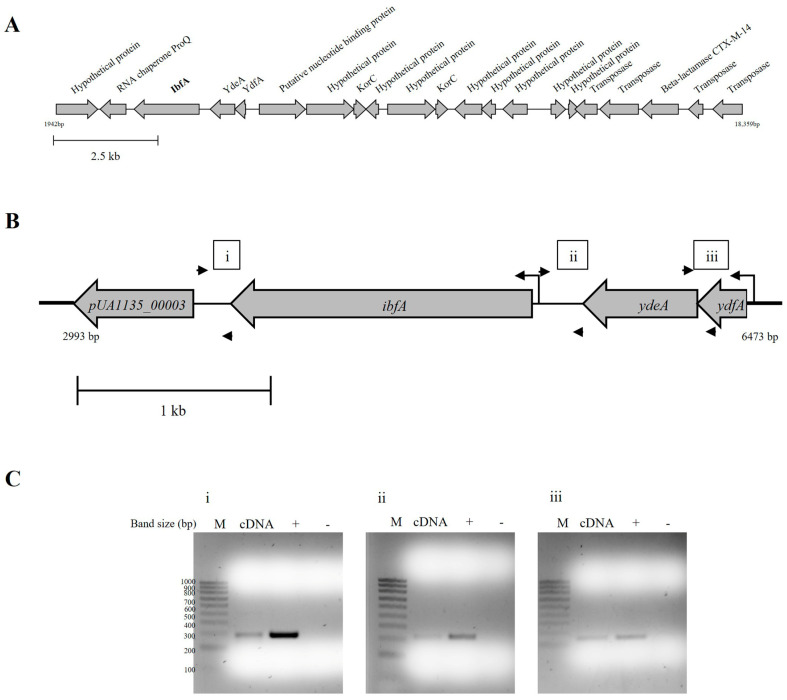
Identification of the transcriptional unit containing *pUA1135_00003*, *ibfA*, *ydeA* and *ydfA* genes encoded in the variable region of pUA1135 plasmid. (**A**) Schematic representation of the variable region contained between nucleotides 2000 (*repA* gene) and 18,500 (*parA* gene). Annotations of each gene correspond to their predicted protein product. (**B**) Detail of the regions detected by RT-PCR for the identification of the transcriptional unit. Small arrows indicate the primers used for RT-PCR, while the large arrows signify the predicted promoters from the transcriptional unit. (**C**) Agarose gel electrophoresis of the products obtained after RT-PCR of the intergenic regions between (i) *pUA1135_00003* and *ibfA*, (ii) *ibfA* and *ydeA*, and (iii) *ydeA* and *ydfA*. M represents the NZYDNA Ladder V marker (NZYTech), cDNA corresponds to the cDNA sample obtained through RT-PCR of the intergenic regions, + indicates a PCR positive control with genomic DNA, and − the PCR negative control.

The variable region of pUA1135 plasmid comprises mainly genes encoding hypothetical proteins, alongside an RNA chaperone, a putative nucleotide-binding protein, a β-lactamase flanked by transposases, two *korC* genes, and homologs of the *ibfA*, *ydeA*, and *ydfA* genes (Figure 2A). Specifically, we found that the DNA region located between positions 3805 bp and 5361 bp in this plasmid, which corresponds to the *ibfA* gene, exhibits a 97.11% and 97.50% sequence identity at the gene and protein levels, respectively, with the *ibfA* gene encoded by the R64 plasmid [34], which was previously reported to interfere with the multiplication of several unrelated phages, including λ, P22 and T7 [30]. Furthermore, using PADLOC [35], we found that *ibfA* was homologous to the uncharacterized HEC-04 defense system, which displays antiviral activity against T4, T5 and several other coliphages [36]. Considering the high sequence similarity between the UAB_Phi20 and P22 phages [27], we hypothesized that the *ibfA* gene encoded in pUA1135 plasmid could be involved in UAB_Phi20 interference. This gene is 1557 bp in length and is located between the *pUA1135_00003* (encoding the RNA chaperone ProQ) and the *ydeA* genes (Figure 2B).

Given their identical orientation, it was possible that the genes *pUA1135_00003*, *ibfA*, *ydeA* and *ydfA* comprised a transcriptional unit. Using BProm [37], we identified a putative promoter sequence for this transcriptional unit, located 103 bp upstream of the *ydfA* gene, with the transcription start site being at 69 bp from the translation start site (TLS). Furthermore, our bioinformatic analysis revealed the presence of a putative promoter for the *ibfA* gene, located 156 bp upstream of its TLS, with a transcription start site at 121 bp from the start of the gene. To confirm that *ibfA* is part of a transcriptional unit, we conducted an RT-PCR analysis on total RNA from the *S*. Typhimurium IC5 variant containing pUA1135. The positive amplification of a DNA fragment for the junction region between each pair of genes suggested that all genes in the cluster constituted an operon transcribed in a polycistronic manner (Figure 2C).

The *ibfA* gene codes for a 518 aa-long protein. HHpred analysis revealed two distinct regions within IbfA. The first region comprised amino acids 1 to approximately 300. This region shows homology to an ATP-binding domain present in several types of nuclease proteins, including endonucleases from the overcoming lysogenization defect family [38,39], the chromosome partitioning protein SMC [40], and other proteins with ATPase components. This would suggest that IbfA possesses an ATPase or ATP-binding domain. The second region (from approximately amino acid 300 to the end of the sequence) shows similarity to the TOPRIM (topoisomerase-primase) domain, which is usually involved in DNA or RNA binding. Furthermore, a sequence-based search with the entire IbfA sequence against the AlphaFold database [41,42] yielded six proteins annotated as members of the AAA-family ATPases or ATP-binding cassette domain-containing proteins, showing a 100% identity match. However, none of these proteins have an experimentally determined function or structure.

To validate the hypothesis of *ibfA* gene interference with the UAB_Phi20 bacteriophage multiplication cycle, the region encompassing the *pUA1135_00003*, *ibfA*, *ydeA* and *ydfA* operon, as well as the individual *ibfA* gene, were deleted from the pUA1135 plasmid. Subsequently, we examined UAB_Phi20 infection on each deletion mutant. The results revealed that both deletions restored the high efficiency of UAB_Phi20 infection on IC5, with EOP values exceeding 0.5 [43] in both mutants (Table 1). To further corroborate this finding, the *ibfA* gene was cloned, along with its own promoter, into the low-copy-number pBAD33 vector, positioning it opposite the arabinose promoter. The construction was then introduced into the previous deletion mutants, and the EOP of the UAB_Phi20 bacteriophage was determined for the resulting strains. Our data revealed that cloning the *ibfA* gene into this vector resulted in a low efficiency of phage infection, with an EOP value of 0.05 (Table 2). Similarly, the entire operon was cloned into the pBAD33 vector and introduced into the IC5 Δ *pUA1135_00003*, *ibfA*, *ydeA*, *ydfA* mutant, yielding results consistent with those mentioned above (Table 2).

### 2.2. ibfA Impacts Phage Productivity Without Impairing Bacterial Growth Kinetics

To investigate the process or processes of the phage multiplicative cycle in which the *ibfA* gene interferes, we obtained the ATCC14028 Rif^R^/pBAD33::*ibfA* strain (*ibfA*+) by introducing the plasmid pBAD33::*ibfA* into the parental strain ATCC14028 Rif^R^. As expected, the infection of this strain by the UAB_Phi20 phage exhibited low efficiency, with an EOP value of 0.05 ± 0.01. However, neither the bacterial growth kinetics (Figure S1) nor the adsorption constant of UAB_Phi20 phage in this strain (1.2 × 10^−8^ ± 5.0 × 10^−9^ mL/min), showed significant variations compared to its parental strain (1.3 × 10^−8^ ± 1.7 × 10^−9^ mL/min). Once the suitability of the *ibfA*+ strain was verified, we proceeded to investigate the role of *ibfA* in phage interference using this strain.

**Figure 3:**
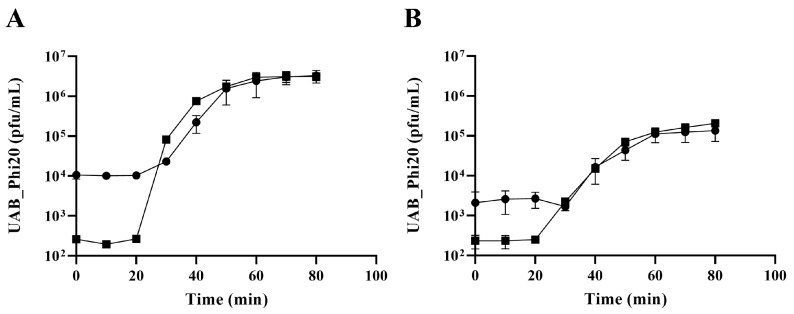
Doermann and one-step growth curves of UAB_Phi20 infection. (**A**) Infection of ATCC14028 Rif^R^/pBAD33 strain; (**B**) infection of ATCC14028 Rif^R^/pBAD33::*ibfA* strain. (●), untreated cultures; (■), chloroform-treated cultures. Error bars indicate standard deviation calculated from three independent experiments.

**Figure 4:**
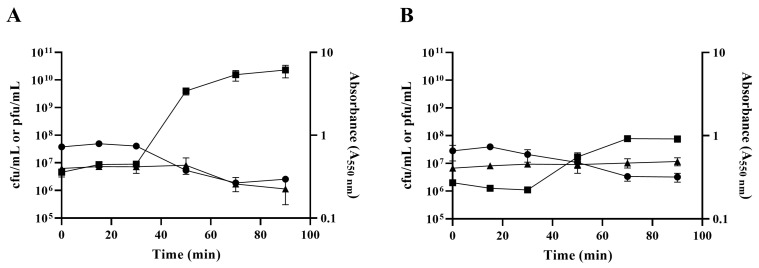
UAB_Phi20 infection kinetics in the absence and presence of *ibfA*. (**A**) Infection curves of UAB_Phi20 upon infection of ATCC14028 Rif^R^/pBAD33 strain; (**B**) infection curves of UAB_Phi20 upon infection of ATCC14028 Rif^R^/pBAD33::*ibfA* strain. (●), Bacterial viable count (cfu/mL); (▲), absorbance (A_550_ nm); (■), UAB_Phi20 concentration (pfu/mL). Error bars represent standard deviation calculated from three independent experiments.

Doermann and one-step growth curves showed that both *ibfA*− and *ibfA*+ strains exhibited similar eclipse and latent periods of 20 and 30 min, respectively, for UAB_Phi20 infection (Figure 3). However, in the presence of *ibfA*, the phage’s burst size was significantly reduced (44.7 ± 7.8 pfu/cell vs. 230 ± 12.1 pfu/cell), coinciding with the smaller plaque size observed in *ibfA*+. Furthermore, the infection kinetics of UAB_Phi20 in *ibfA*+ liquid cultures (Figure 4) showed an altered pattern compared to *ibfA*−. The absorbance of cultures remained stable throughout the course of infection in the presence of *ibfA*, but decreased in its absence. Moreover, the presence of the *ibfA* gene gave rise to a reduction of approximately 1.5 log_10_ in phage production (Figure 4). Finally, the ECOI values of UAB_Phi20 in the presence and absence of this gene were 0.16 and 1, respectively.

### 2.3. Impact of the ibfA Gene on the Multiplicative Cycle of UAB_Phi20 Bacteriophage

Subsequent experiments sought to elucidate which phase of the phage multiplicative cycle after injection of DNA was affected by the presence of the *ibfA* gene.

**Figure 5:**
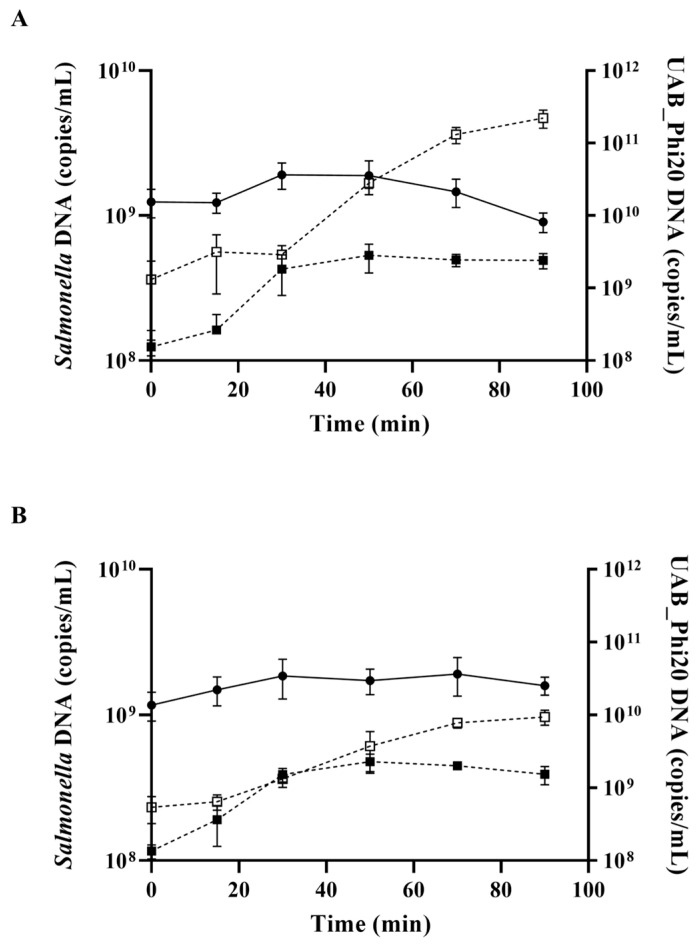
Genome replication of the UAB_Phi20 phage in the absence and presence of *ibfA*. qPCR quantification of UAB_Phi20 inside ATCC14028 Rif^R^/pBAD33 (**A**) and ATCC14028 Rif^R^/pBAD33::*ibfA* (**B**) strains. (●) Bacterial genome copies/mL; bacteriophage genome copies/mL inside (■) and outside (□) *Salmonella* cells. Time 0 was defined as 15 min after phage adsorption of synchronized cultures. Data correspond to three biological replicates with error bars showing standard deviation.

First, the replication of the phage genome was studied after infection of synchronic bacterial cell cultures. The number of UAB_Phi20 DNA copies inside cells increased by 1-log_10_ 30 min after phage adsorption to the cell, indicating active phage genome replication. This increase was independent of the *ibfA* gene (Figure 5). Thereafter, the intracellular UAB_Phi20 DNA copies stabilized in the absence of the *ibfA* gene, while a non-significant decrease was observed in the presence of *ibfA* 50 min after phage adsorption. In contrast, the number of extracellular UAB_Phi20 DNA copies remained constant until 30 min after phage adsorption, after which a significant increase in extracellular phage DNA copies was observed. This increase denotes the release of newly formed phage particles following intracellular replication and assembly. However, this increase was approximately two-log_10_ in the absence of the ibfA gene, compared to only about 1-log_10_ in its presence (Figure 5).

**Figure 6:**
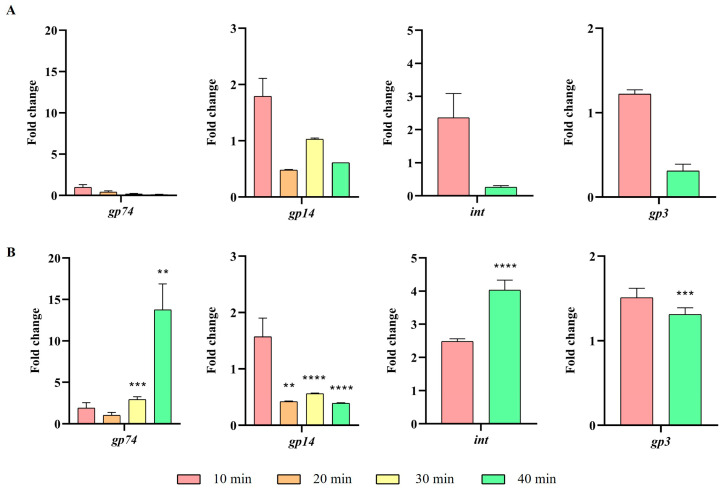
Expression levels of early UAB_Phi20 genes. (**A**) Expression in the absence of *ibfA* and (**B**) in the presence of this gene after phage adsorption. RT-qPCR quantification analysis was performed using the E-method, with time 0 as a calibrator and 16S rRNA as the reference gene. The values represent the geometric mean and the standard deviation of the mean of three independent experiments performed in duplicate. ** *p* < 0.01, *** *p* < 0.001 and **** *p* < 0.0001.

**Figure 7:**
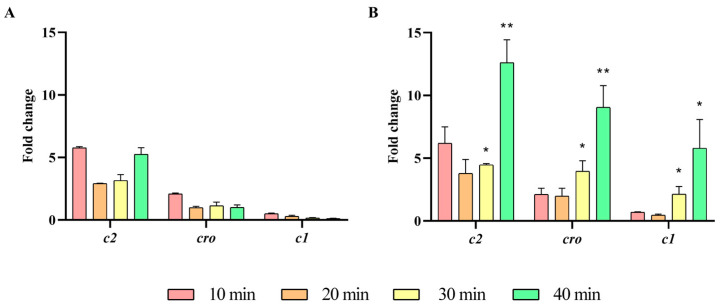
Expression of UAB_Phi20 *immC* region genes. (**A**) Expression in the absence of *ibfA* and (**B**) in the presence of this gene after phage adsorption. RT-qPCR quantification analysis was performed using the E-method, with time 0 as a calibrator and 16S rRNA as the reference gene. The values represent the geometric mean and the standard deviation of the mean of three independent experiments performed in duplicate. * *p* < 0.05 and ** *p* < 0.01.

Next, the expression of UAB_Phi20 early genes was studied by RT-qPCR in the presence and absence of the *ibfA* gene, following infection of synchronic bacterial cultures. Specifically, the early genes studied were *gp74*, *int*, *gp14*, and *gp3*, which encode the early antitermination, integrase, late antitermination, and replicative DNA helicase proteins, respectively, and whose expression is regulated from the P_R_ or P_L_ promoters (Figure S2). Our results revealed that expression of three out of four early genes was significantly higher at 30 min and 40 min in the presence of the *ibfA* gene, except for the *gp14* gene, whose expression was significantly lower (Figure 6). We also examined the expression of the *cro*, *c2* and *c1* early genes of the *immC* region (Figure S3B). These genes encode the Cro repressor, C2 repressor and protein C1, which are involved in lysogeny establishment in temperate bacteriophages such as P22. Results also demonstrated an increase in the expression of these genes in the presence of the *ibfA* gene, similar to the trend observed for the other early genes (Figure 7). Given that transcription of the *c2* gene was not initially expected due to the reported absence of a C1 protein binding site in the P_RE_ promoter of UAB_Phi20 bacteriophage [27], the genome sequence of this bacteriophage was reanalyzed, and a recognition site for the C1 protein was identified on the P_RE_ promoter, albeit with a single nucleotide change (TT**G**CN6TTGT) compared to the corresponding site (TT**T**CN6TTGT) in the P22 bacteriophage.

**Figure 8:**
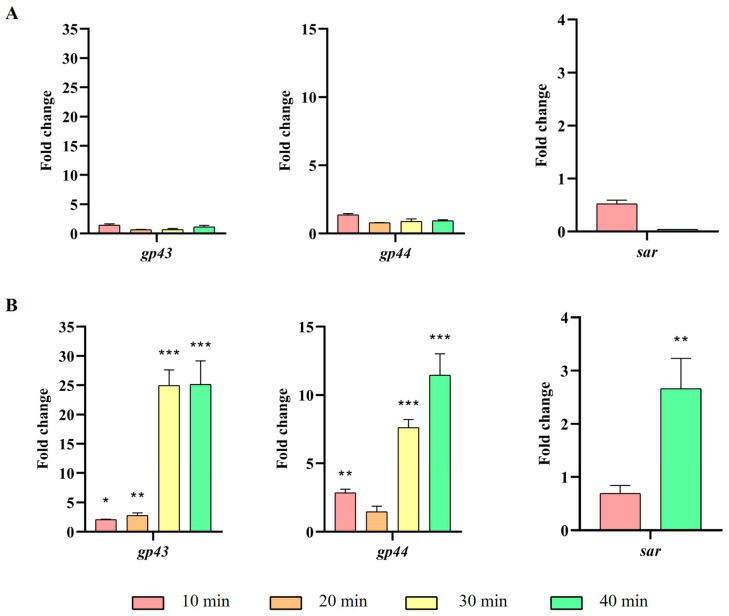
Expression of UAB_Phi20 *immI* region genes. (**A**) Expression in the absence of *ibfA* and (**B**) in the presence of this gene after phage adsorption. RT-qPCR quantification analysis was performed using the E-method, with time 0 as a calibrator and 16S rRNA as the reference gene. The values represent the geometric mean and the standard deviation of the mean of three independent experiments performed in duplicate. * *p* < 0.05, ** *p* < 0.01 and *** *p* < 0.001.

A sizeable proportion of P22-like phages harbor a genomic region known as *immI*, which includes the *arc*, *ant* and *sar* early genes. These genes encode the Arc repressor, responsible for regulating the expression from the P_ANT_ promoter, the Ant antirepressor of the C2 repressor, and the small antisense RNA (Sar), respectively (Figure S3C). In line with the observations made for early genes, an increase in the expression levels of these genes was observed in the presence of *ibfA* (Figure 8).

**Figure 9:**
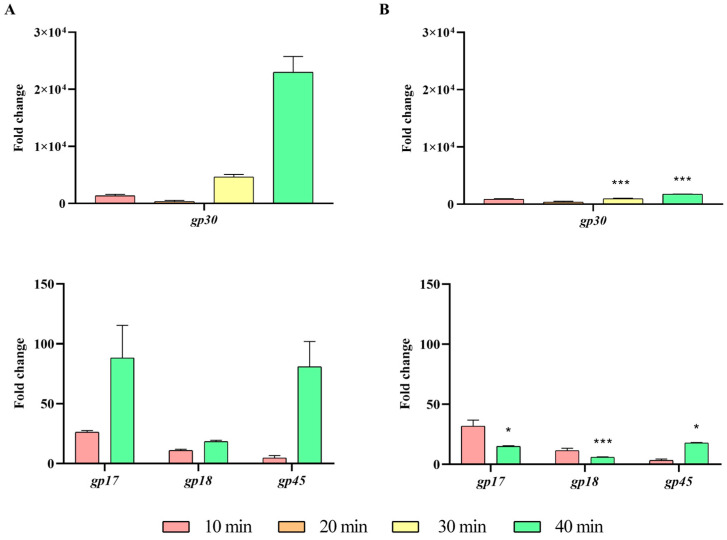
Expression levels of late UAB_Phi20 genes. (**A**) Expression in the absence of *ibfA* and (**B**) in the presence of this gene after phage adsorption. RT-qPCR quantification analysis was performed using the E-method, with time 0 as a calibrator and 16S rRNA as the reference gene. The values represent the geometric mean and the standard deviation of the mean of three independent experiments performed in duplicate. * *p* < 0.05 and *** *p* < 0.001.

Finally, the expression of the late genes *gp30*, *gp17*, *gp18*, and *gp45*, which encode the coat, holin, lysozyme, and tailspike proteins, respectively, was also studied (Figure S4). The results indicated a significant decrease in the expression of these four genes after 30 and 40 min in the presence of the *ibfA* gene (Figure 9).

### 2.4. Visualization of UAB_Phi20 Virions by Electron Microscopy

**Figure 10:**
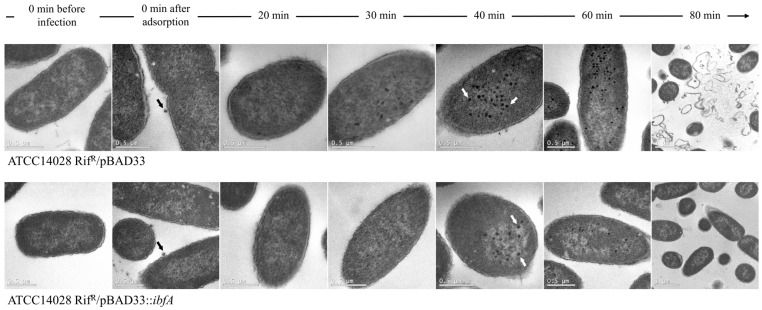
Transmission electron micrographs of ultrathin sections from ATCC14028 Rif^R^/pBAD33 and ATCC14028 Rif^R^/pBAD33::*ibfA* cells after infection with UAB_Phi20 phage at different times. Black and white arrows indicate adsorbed phages at time 0 min after adsorption, and fully formed capsids at 40 min, respectively. All image scales are 0.5 µm except at 80 min, where they are 1 µm.

The assembly of UAB_Phi20 virions in the presence and absence of the *ibfA* gene was examined at an ultrastructural level using cellular thin sections observed through TEM. This was carried out over time after infection of synchronic cultures, as depicted in Figure 10. Initially, only a few bacteriophages were observed attached to both types of bacterial cells after phage adsorption (time 0). Intracellular virions were first detected in the absence of the *ibfA* gene at 30 min. By 40 min, fully formed capsids were distinguishable inside infected cells, albeit with a notable difference in the number of viral particles between the *ibfA*+ and *ibfA*-strains (Table 3). Similarly, the percentage of sections containing virions relative to the total number of recorded sections was significantly lower in the presence of the *ibfA* gene (Table 3). These differences became more pronounced at 60 min, with 88.8% of sections showing virions when the *ibfA* gene was absent, compared to 3.6% in its presence (Table 3). Furthermore, by 80 min, complete lysis of cells lacking the *ibfA* gene was evident, whereas barely any lysis could be observed in cells containing this gene (Figure 10).

### 2.5. Distribution of ibfA-Related Genes in Prokaryotic Genomes

The dissemination of *ibfA* among prokaryotic genomes was investigated through a BLASTP search of IbfA against the nr database, filtering results with an 85% query coverage threshold, which yielded 2416 hits. The identified proteins belonged exclusively to the *Enterobacteriaceae* family, with the majority originating from *Salmonella enterica* (1388/2416; 57.5%) and *Escherichia* (933/2416; 38.6%). To ascertain whether IbfA homologs were also present outside *Enterobacteriaceae*, another BLASTP search was conducted against the clustered nr database, filtering results for 85% query coverage and an e-value of 1 × 10^−5^. This search yielded 214 hits that exhibited a much broader distribution across bacterial and archaeal taxonomy. Among these proteins, those identified in complete chromosome or plasmid sequences were selected and phylogenetic inference was performed on the IbfA sequences. (Figure S5). The analysis revealed that IbfA from pUA1135 clustered with IbfA from other *Enterobacteriaceae* species and was predominantly found amongst other plasmid-encoded IbfA homologs. Conversely, the majority of the IbfA homologs from other phylogenetic lineages were located within chromosomes.

## 3. Discussion

In previous work [25], we identified large conjugative plasmids mediating bacteriophage interference mechanisms. These plasmids were found in *S*. Typhimurium ATCC14028 Rif^R^ variants isolated from a phage therapy study performed on broiler chickens experimentally contaminated and treated with a three-phage cocktail containing the UAB_Phi20, UAB_Phi78 and UAB_Phi87 phages. Among those variants was IC5, harboring the IncI1α plasmid pUA1135, which was responsible for interference of the P22-like UAB_Phi20 bacteriophage. This prompted us to determine the gene or genes responsible for UAB_Phi20 interference and to elucidate the potential novel molecular mechanism behind it. In this study, we demonstrate that the *ibfA* gene, located on plasmid pUA1135, is responsible for interference of UAB_Phi20, and we describe the mechanism through which IbfA leads to this defense.

Defense systems are commonly encountered within mobile genetic elements, including conjugative plasmids [44,45], and often within regions of high variability therein [33]. Our initial analysis of the pUA1135 sequence revealed the presence of a hypervariable region situated between *repA* and *parA* genes (Figure 2A). Given its variability, we hypothesized that the segment of pUA1135 spanning from positions 2000 bp to 18,500 bp could contain the gene or genes responsible for the mechanism of defense against UAB_Phi20. Indeed, deletion of a full transcriptional unit, comprising the *pUA1135_00003*, *ibfA*, *ydeA*, and *ydfA* genes (Figure 2), or solely the *ibfA* gene, led to the restoration of susceptibility to UAB_Phi20 in the IC5 variant (Table 1), underscoring the essential role of *ibfA* in UAB_Phi20 interference. It is notable that complementation of this gene did not fully reverse the interference with UAB_Phi20 (Table 2). We speculate that perhaps additional gene(s) encoded in the pUA1135 plasmid, and located outside the *ibfA* transcriptional unit, may be required for the full functionality of *ibfA* in the interference phenotype of UAB_Phi20, or that the *ibfA* copy number mediated by the pUA1135 plasmid is essential for achieving robust interference.

The *ibfA* gene identified in pUA1135 is homologous to the *ibfA* gene encoded in the R64 plasmid [34], an IncI1 incompatibility group plasmid that hinders infection by coliphages BF23, PhiI and λ, as well as *S*. Typhimurium phages P22 and St [29,30,46], and which has more recently been associated with changes in phage type [28]. It must be noted that the ColIb plasmid also harbors a gene annotated as *ibfA* [47], but this gene shares no sequence identity with *ibfA* from pUA1135. Furthermore, we found that *ibfA* is also homologous to the recently described HEC-04 defense system [36]. The HEC systems, referred to as Hma (helicase, methylase, ATPase)-embedded-candidate systems, are genes found between Hma system genes that have been confirmed to confer phage resistance [48]. In particular, HEC-04 is an ABC ATPase protein fused to a TOPRIM domain that confers defense against *Escherichia coli* phages T4 and T5, albeit not against T7 or λ. Our bioinformatic analysis of the functional domains of the IbfA protein encoded in the pUA1135 plasmid showed that this protein contains both ATPase and TOPRIM domains. Several phage defense systems are known to operate through the association of ATPase and TOPRIM domains, as exemplified by the two-protein PARIS [49] and Gabija [50] systems. Furthermore, phage interference attributed to cellular ATP depletion has also been described [51]. Consequently, we analyzed the effect of the *ibfA* gene on ATP concentration in infected and non-infected cultures. Infection with UAB_Phi20 reduced ATP levels in the parental strain due to the ATP-dependent processes involved in phage formation [52]. However, this ATP depletion did not occur in the *ibfA*+ strain, indicating that IbfA is not an ATPase. Instead, the lower burst size of UAB_Phi20 when infecting *ibfA*-carrying cells (Figure 3), suggested that IbfA disrupted phage-particle formation.

IbfA clearly impacts the productivity of UAB_Phi20 infection, as evidenced by the reduced EOP, ECOI and plaque size. Lower productivity could result from an extended latent period or a decrease in burst size [53]. Our results clearly showed that the latent and eclipse periods were not altered by the presence of *ibfA* gene (Figure 3). Instead, we observed a significant reduction in UAB_Phi20 burst size (Figure 4). TEM imaging on cellular cross sections of infected cells corroborated that the number of observed infected cells and the number of viral particles assembled inside *ibfA*+ cells was significantly lower than in the absence of this gene (Figure 10 and Table 3). In addition to the EOP, ECOI and burst size values, the infection kinetics of the UAB_Phi20 phage also pointed to an *ibfA*-mediated interference mechanism reminiscent of an abortive infection, because cellular viability decreased without observable cell lysis (Figure 4).

We also analyzed whether the *ibfA*-mediated interference affected replication or transcription of the phage genome in synchronized bacterial cultures. The quantification by qPCR of the UAB_Phi20 DNA inside and outside *Salmonella* cells, as well as *Salmonella* intracellular DNA, showed phage genome replication beginning 30 min after bacteriophage adsorption in cells that did not contain the *ibfA* gene (Figure 5A). After this, UAB_Phi20 DNA began to increase outside the cell (Figure 5A), coinciding with the observed latent period of the phage (Figure 3). In the presence of *ibfA*, UAB_Phi20 DNA inside *Salmonella* mirrored the pattern observed in the parental strain (Figure 5B), suggesting successful DNA injection and unaffected replication of the UAB_Phi20 genome. However, extracellular phage DNA levels were approximately 1.5-log_10_ lower, in agreement with the reduced burst size of the phage in presence of *ibfA*.

Following genome replication, a pivotal phase in the phage life cycle involves the transcription of genes to facilitate the production of essential structural proteins for the assembly of new virions as well as proteins involved in cell lysis. The quantification by RT-qPCR of the expression of early (*gp74*, *int*, and *gp3*) genes, those in the *immC* region (*cro*, *c2* and *c1*), and those in the *immI* region (*gp43*, *gp44* and *sar*), demonstrated that their expression increases significantly at 30 min and 40 min in the presence of *ibfA* (Figure 6, Figure 7 and Figure 8), except for the *gp14* early gene, whose expression was significantly lower (Figure 6). This gene codes for the late antitermination protein, which facilitates antitermination of the transcript generated from the P_LATE_ promoter [54] and is located at the distal end of the P_R_ transcript (Figure S2). Furthermore, it is negatively regulated by a small antisense RNA expressed from the P_A23_ promoter (Figure S2), which is in turn positively regulated by C1 [55]. Consequently, the increased expression of the early gene *c1* at 30 and 40 min leads to a decreased expression of the late antiterminator. Additionally, the diminished expression of *gp14* will lead to reduced expression of the genes controlled by P_LATE_ (*gp30*, *gp17*, *gp18* and *gp45*; Figure S4), encoding phage structural and cellular lysis proteins, at 30 and 40 min, as was detected (Figure 9). These results correlate with the decreased burst size, the infection kinetics (Figure 4) and the almost nonexistent lysis observed through TEM in *ibfA*+ cells (Figure 10).

The *immI* region of phage P22 has been amply studied [56,57,58]. In UAB_Phi20, this region contains the *arc*, *ant* and *sar* early genes. We hypothesize that the significantly increased expression of the early genes in the presence of *ibfA* at 30 and 40 min post-adsorption is related to the increase in the expression of the *ant* gene, which encodes the Ant antirepressor that inhibits C2, repressor of the lytic cycle [54]. Both *arc* and *ant* genes are transcribed from the P_ANT_ promoter (Figure S3C), which is self-repressed by the Arc protein during the lytic cycle [59]. Furthermore, both genes are also transcribed from the P_LATE_ promoter (Figure S3), but this transcription would be low in the presence of *ibfA* as it occurs for the other late genes. On the other hand, the *sar* gene encodes a small antisense RNA transcribed from P_sar_ and inhibits *arc* translation from the P_LATE_ promoter [59,60]. Therefore, we hypothesize that *ibfA* could be directly or indirectly hampering the function of the Arc protein. As a result, an intracellular increase in the antirepressor Ant would be expected and the equilibrium of C2 and Cro concentration, responsible for the control of lysis/lysogeny decision, would be disrupted, causing an increase in early gene expression from P_R_ and P_L_. In fact, a similar phenotype has been previously described by the P22 phage [61], where an Arc-mutant underproduced late proteins and overproduced early proteins, though the author did not offer a definitive explanation for the observed phenotype. The underexpression of the late proteins agrees with the characteristics commented in this work about the *ibfA*-mediated interference with the multiplicative cycle of the UAB_Phi20.

Phylogenetic analysis of IbfA homologs revealed a wide distribution of this protein across the Bacteria and Archaea domains. Close homologs of pUA1135 IbfA are predominantly plasmid borne and widely disseminated within the *Enterobacteriaceae* (Figure S5), suggesting extensive dissemination through lateral gene transfer, a hallmark of plasmid-borne phage defense systems [62]. In contrast, more distant homologs of pUA1135 IbfA are typically chromosomally encoded, and their phylogenetic distribution points to recurrent transfer events followed by chromosomal consolidation in different lineages. This complex evolutionary history suggests that IbfA homologs may have host-beneficial roles other than phage defense. Other proteins that, like IbfA, contain both ATPase and TOPRIM domains, such as type II topoisomerases, can couple the energy from ATP hydrolysis to the manipulation of DNA topology, and therefore play crucial roles in processes like DNA replication, repair, and chromosome segregation [63]. Then again, the likely dissemination of distant IbfA homologs through lateral gene transfer events suggests that they may have first entered the identified lineages as a defense mechanism against *Lederbergvirus*-related phages, and were later co-opted to perform other functions in the cell.

We have demonstrated that *ibfA* encodes a defense mechanism that reduces the infectivity and productivity of UAB_Phi20, as evidenced by a significant decrease in EOP, ECOI, and burst size, occurring without observable bacterial lysis but with reduced cellular viability. Furthermore, *ibfA* interferes with the UAB_Phi20 genome transcription by enhancing the expression of early genes, thereby disrupting the Cro/C2 regulatory balance. This imbalance leads to a reduction in the expression of structural virion and lysis-associated proteins, ultimately resulting in abortive infection. This mechanism would be extensive to P22-type phages. Moreover, despite the uncertain evolutionary origin of *ibfA*, we have found widespread distribution of this gene among prokaryotic genomes, both chromosomal and plasmid-borne (Figure S5), a characteristic of phage defense systems [3,9,10,23,33,44,45]. Therefore, we suggest that *ibfA* might possess additional functions beyond phage defense that would be beneficial for the host bacterial cells. Furthermore, its presence across a non-standard bacterial phylogeny suggests that *ibfA* may disseminate through bacterial populations via plasmids and has been integrated into bacterial chromosomes through diverse events of bacterial evolution.

## 4. Materials and Methods

### 4.1. Bacterial Strains

The bacterial strains and plasmids used in this study are described in Table S2. The parental strain used to determine the efficiency of plating (EOP) and study phage defense mechanisms was a spontaneous mutant of *Salmonella enterica* serovar Typhimurium ATCC14028 Rif^R^, obtained by us. The *S*. Typhimurium IC5 variant [25] was used in the deletion and complementation experiments of genes involved in phage defense mechanisms. Finally, the strain *Escherichia coli* DH5α was used for cloning phage defense genes into pBAD33 plasmid [64]. ATCC14028 Rif^R^, IC5, and the derived strains were cultured on Luria–Bertani (LB) liquid (10 g L^−1^ NaCl, 5 g L^−1^ yeast extract, 10 g L^−1^ tryptone) or agar LB with 15 g L^−1^ agar) media supplemented with rifampicin (75 µg/mL). Strains containing pBAD33 plasmid were grown in media supplemented with 34 µg/mL of chloramphenicol. All bacterial culture plates were incubated for 18 h at 37 °C. Phage infection experiments were performed with the phage UAB_Phi20 (accession number: NC_031019) [26,27].

### 4.2. Phage Lysate Production

Phage lysates were obtained following a previously described protocol [65] and filtered through 0.45 µm pore size polyether sulfone (PES) membranes (Millex^®^-HP, Merck KGaA, Darmstadt, Germany). Phage titration was performed by plating ten-fold serial dilutions onto LB plates using the double agar layer method [66].

### 4.3. Bacterial Growth Kinetics

*Salmonella* liquid LB cultures were grown for 18 h at 37 °C with agitation. Afterwards, cultures were diluted (1:100) and incubated at 37 °C monitoring the absorbance at 550 nm (A_550nm_) using the spectrophotometer Genesys 10S UV-Vis (Thermo Fisher Scientific, Massachusetts, MA, USA) at 30 min or 1 h intervals. In addition, the concentration of viable cells as colony-forming units per mL (cfu/mL) was determined at each interval. At least two independent experiments were performed to obtain the standard deviations.

### 4.4. EOP, Phage Adsorption Constant, Efficiency of Center of Infection (ECOI), and Burst Size Determination

For EOP determination, bacterial cultures were incubated at 37 °C for 18 h in LB medium until they reached the stationary phase. One hundred µL of the bacterial culture (1 × 10^8^ cfu mL^−1^) were mixed with 100 µL of ten-fold serial dilutions of UAB_Phi20 lysate in 0.7% LB molten agar. The mixture was plated in LB agar by the double agar layer method [64]. The number of plaque-forming units (pfu) was counted after 18 h incubation at 37 °C and the EOP was calculated as described previously [43]. Three replicates were performed, and the standard deviations were calculated.

Measurement of the adsorption rate constant of UAB_Phi20 was carried out as described previously [67]. Both the center of infection (COI) and the burst size were determined as described in Moineau et al. [68] and Kropinski [69] with some modifications. Briefly, *Salmonella* cultures incubated at 37 °C for 18 h were diluted 1/100 and incubated at 37 °C until they reached mid-log phase. Cultures were then adjusted to A_550nm_ = 0.4 and incubated for 5 min at 37 °C with shaking at 60 r.p.m. Infection was performed at a multiplicity of infection (MOI) of 0.1 pfu/cfu, and the phage was allowed to adsorb for 15 min at 37 °C without shaking. Two serial 1/100 dilutions were carried out and 100 µL of the 10^−4^ dilution were plated onto a lawn of ATCC14028 Rif^R^ (parental sensitive strain) by the double agar layer method [66]. The number of plaques determined the COI formed on the test strain. The ECOI was calculated by dividing the number of COI from the test strain by the number of COI from the parental strain ATCC14028 Rif^R^ (sensitive strain). A control of correct adsorption of the phage was performed by mixing 1 mL of the 10^−4^ dilution to 100 µL of ice-cold chloroform. The mixture was centrifuged for 1 min at 6797× *g*, and 100 µL of the supernatant were plated by the double layer method using the ATCC14028 Rif^R^ parental strain [66]. To determine the burst size, the 10^−4^ dilution was serially diluted up to a 10^−7^ dilution. One hundred µL samples were collected from the appropriate dilution every 10 min up to 125 min of phage infection, mixed in 0.7% LB molten agar containing 100 µL of ATCC14028 Rif^R^ and plated in LB agar by the double layer method [66]. Burst size on a test strain was calculated by dividing the final concentration of phage to the resulting number of the subtraction of the adsorption control to the initial concentration of phage [68,69]. Experiments were performed at least three times, and the standard deviations were calculated.

### 4.5. One-Step Growth

Experiments of one-step growth were performed as described [70] with some modifications. The desired *Salmonella* cultures were grown in LB medium for 18 h at 37 °C. Afterwards, cultures were diluted and incubated at 37 °C until an A_550nm_ of 0.4 was reached. Cultures were synchronized with potassium cyanide (KCN) 10 mM (Merck Life Science S.L.U., Madrid, Spain) for 10 min at 37 °C without shaking. Afterwards, they were infected with UAB_Phi20 bacteriophage at a MOI of 1 pfu/cfu and left at 37 °C without shaking for the 15 min required for UAB_Phi20 adsorption. Then, the cultures were centrifuged at 12,880× *g* for 10 min and the pellet resuspended in the same volume of fresh LB. Cultures were serially diluted up to 10^−8^ in the case of ATCC14028 Rif^R^ pBAD33 and 10^−7^ for ATCC14028 Rif^R^ pBAD33::*ibfA* and incubated at 37 °C with shaking at 150 r.p.m. One hundred µL samples were taken every 10 min until 80 min after phage adsorption, and titration was carried out by double agar layer method [66] using ATCC14028 Rif^R^ as host strain without any treatment, to determine the latent period. Furthermore, 100 µL of samples were mixed with 800 µL LB and 100 µL chloroform, vortexed and centrifuged at 6797× *g* for 1 min, and the titration of the supernatant was carried out to release intracellular phages (eclipse period). Three replicates were performed, and standard deviations were determined.

### 4.6. Phage Infection Kinetics

*Salmonella* bacterial cultures infected with UAB_Phi20 were prepared as explained above. Following the 15 min of phage adsorption and subsequent removal of KCN, 1 mL samples were withdrawn at specified intervals and centrifuged at 6797× *g* for 2 min. The titration of the supernatant was carried out by the double agar layer method [66] to quantify the produced phage. The pellet was washed with 1 mL of cold NaCl (0.9%) and centrifuged at 6797× *g* for 2 min. The resulting pellet was then resuspended in 1 mL cold NaCl (0.9%) and serially diluted to plate the viable cells on LB agar plates. Simultaneously, at the same time points, 800 µL aliquots were collected from the cultures and directly mixed with 200 µL of 10% formaldehyde, and at the end of the assay the A_550nm_ was determined. Three replicates were performed, and standard deviations were determined.

### 4.7. Deletion of ibfA Through One-Step Inactivation

A λ-Red recombinase strategy [71] was performed to delete *ibfA* or the *ibfA*-containing transcriptional unit from pUA1135 (NCBI accession number: MW590592.1) in the IC5 variant. Briefly, *Salmonella* cells were made electrocompetent and the pKOBEG plasmid [72] was electroporated and selected on LB agar plates containing chloramphenicol (34 µg/mL). Simultaneously, the kanamycin resistance cassette from pKD4 plasmid was obtained by PCR using Phusion™ High-fidelity DNA polymerase (Thermo Scientific™, Vilnius, Lithuania) and using primers with homology tails matching the desired region in the pUA1135 plasmid (Table S3). The resulting linear product was purified with NzyGelpure kit (NZYTech, Lisboa, Portugal) and introduced by electroporation in target pKOBEG-containing cells. Transformant cells were selected in LB agar plates containing 50 µg/mL kanamycin and 34 µg/mL chloramphenicol and incubated at 30 °C overnight. The kanamycin cassette insertion was confirmed by PCR and sequencing (Macrogen, Amsterdam, The Netherlands) using the appropriate primers (Table S3). The pKOBEG plasmid was cured by incubating at 42 °C and clones that were kanamycin resistant and chloramphenicol susceptible were selected.

### 4.8. Transcriptional Analysis of ibfA Operon

RNA extraction was carried out from *Salmonella* cultures using the RNeasy Mini kit (Qiagen, Hilden, Germany) following manufacturer’s instructions with a previous lysis step with lysozyme (50 mg/mL) in TE buffer (10 mM Tris-HCl, pH 7.5, 1 mM EDTA, pH 8.0) at 37 °C, vortexing every 2 min for 10 min. DNA removal was accomplished with the TURBO DNA-free kit (Invitrogen, Vilnius, Lithuania) as described by the manufacturer with modifications. Briefly, 90 µL samples were incubated with 1X TURBO DNase™ Buffer and 8 units of TURBO DNase™ Enzyme for 45 min at 37 °C. This treatment was performed twice. Enzyme inactivation was carried out according to manufacturers’ instructions. RNA sample cleanup was carried out using the RNeasy Mini kit (Qiagen, Hilden, Germany).

Reverse transcription of DNase-treated RNAs (1 μg) was carried out with NZY First-Strand cDNA Synthesis Kit, with separate oligos (NZYTech, Lisboa, Portugal) following the manufacturer’s conditions. Briefly, the first step reaction (8 µL) included 1 µg of RNA, appropriate primers (2 µM; see Table S3), and 1X annealing buffer. Samples were incubated at 65 °C and then placed on ice. The second-step reaction (20 µL) contained 1X NZYRT Master Mix and 2 μL of NZYRT Enzyme Mix, and was incubated at 55 °C for 30 min, followed by enzyme inactivation at 85 °C for 5 min. A final treatment with 1 µL of NZY RNase H at 37 °C for 20 min was performed to remove RNA bond to cDNA. Subsequent cDNA amplification was carried out by conventional PCR using the Invitrogen^TM^ Taq DNA Polymerase kit (Invitrogen, Carisbad, CA, USA). PCR reactions (20 µL) included 10 μM of each primer (same as above), 1X PCR buffer minus Mg, 10 mM dNTP, 50 mM MgCl_2_, *Taq* DNA Polymerase (5 U/μL), and 2 µL of template cDNA. PCR conditions included an initial denaturation at 95 °C for 5 min; 30 cycles of 95 °C for 30 s, 55 °C for 30 s, and 72 °C for 45 s; followed by a final step at 72 °C for 7 min. The resulting PCR products were visualized by agarose (2%) gel electrophoresis and stained in RedSafeTM Nucleic Acid Staining Solution (iNtRON, Kirkland, WA, USA). DNA molecular weight marker NZYDNA Ladder V marker (NZYTech, Lisboa, Portugal) was used as standard.

### 4.9. Cloning of ibfA onto the Vector pBAD33

The genomic DNA was purified from *S*. Typhimurium IC5, which contains the pUA1135 plasmid, by using the Easy-DNA^TM^ kit (Invitrogen, Carisbad, CA, USA), following the manufacturer’s instructions. Gene *ibfA* or the *ibfA*-containing transcriptional units were PCR amplified from pUA1135 with their own promoter using primers listed in Table S3. The PCR product was purified using NzyGelpure kit (NZYTech, Lisboa, Portugal) and inserted onto the vector pBAD33, previously linearized with KpnI restriction enzyme (New England Biolabs, Inc., Ipswich, MA, USA), using NEBuilder^®^ HiFi DNA Assembly Master Mix (New England Biolabs, Inc., Ipswich, MA, USA). This construction was then transformed into DH5α competent cells by electroporation and selected on LB agar plates supplemented with chloramphenicol (34 μg/mL). The correct cloning of *ibfA* or the transcriptional unit was checked by PCR and sequencing (Macrogen, Amsterdam, The Netherlands) using appropriate primers (Table S3).

### 4.10. Replication of UAB_Phi20 Genome

The quantification of *Salmonella* DNA, and intracellular and extracellular UAB_Phi20 DNA was performed by quantitative real-time PCR (qPCR). Bacterial cultures were prepared as explained above and infected with UAB_Phi20 at a MOI of 1 pfu/cfu. One mL aliquots were withdrawn just after phage adsorption (t: 0 min) and at 15, 30, 50, 70 and 90 min, and centrifuged at 6797× *g* for 2 min. The supernatants were kept at 4 °C and used to quantify extracellular bacteriophage DNA. The pellets were washed once with NaCl (0.9%) and frozen at −80 °C. Once all samples were collected, pellets were defrosted and resuspended in 100 µL TE buffer (10 mM Tris-HCl, pH 7.5, 1 mM EDTA, pH 8.0). Cell lysis was accomplished by temperature treatment (100 °C) for 10 min. Samples were then centrifuged at 6797× *g* for 2 min and the supernatants were kept at −20 °C for quantification of *Salmonella* DNA and intracellular bacteriophage DNA.

qPCR was prepared using 2 µL of template DNA from cell pellets or 2 µL of supernatants, appropriate primers (0.5 µM final concentration) (Table S3), milli-Q water and 1X LightCycler^®^ 480 SYBR Green I Master kit buffer (Roche, Mannheim, Germany) for a final volume of 20 µL. qPCR assays were performed on a Lightcycler 480^®^ Instrument II (LC480, Roche, Basel, Switzerland) with the following cycle conditions: preincubation at 95 °C for 30 s followed by 45 amplification cycles of 3 s at 95 °C, 15 s at 57 °C and 18 s at 72 °C, and a final melting curve up to 95 °C. All qPCR reactions were made in technical duplicates, and non-infected samples were included as negative controls. Total DNA extracted from *S*. Typhimurium ATCC14028 Rif^R^ using the Easy-DNA^TM^ kit (Invitrogen, Carisbad, CA, USA) and UAB_Phi20 DNA purified by the phenol-chloroform protocol [73] were used to generate the respective standard curves. For this, 10-fold gradient dilutions of each DNA were prepared representing 100 to 10^9^ genome copies/µL of templates and tested in triplicate to obtain the copy number per µL from the target genes *gyrB* (*Salmonella*) and helicase (*gp3*, UAB_Phi20) (Table S3). The determination of gene copies according to the size of the bacteria or phage genomes, and the concentration of DNA used, was obtained as previously described [74]. The results in copies/mL of intracellular and extracellular phage DNA and *Salmonella* cells were obtained from imported standard curves as described [74], taking into account the dilution factor of each sample when necessary. Biological triplicates were prepared, and qPCR experiments were performed from two technical replicates to calculate the average copy number per mL, and the standard deviations.

### 4.11. Gene Expression Quantification

To assess the expression of some UAB_Phi20 phage genes and the bacterial gene *ibfA*, reverse transcription-quantitative polymerase chain reaction (RT-qPCR) was performed. Bacterial cultures were prepared as explained above. Uninfected cultures were treated similarly to the infected ones, but without the addition of the phage.

Two samples of 5 mL were collected at 0, 10, 20, 30 and 40 min after phage adsorption and mixed with 1/10 stop solution (1:10 buffered phenol, 9:10 absolute ethanol) as described previously [75], vortexed and placed on ice for total RNA extraction. When all samples were collected, they were centrifuged at 12,857× *g* for 10 min and the supernatant was removed. Pellets were stored at −80 °C until RNA extraction, performed using the RNeasy Mini kit (Qiagen, Hilden, Germany) as described above, except for an additional DNase treatment consisting of the addition of 5 µL of 10X TURBO DNase™ Buffer and 4 units of TURBO DNase™ Enzyme (Invitrogen, Vilnius, Lithuania) that was incubated for 1 h at 37 °C.

One-step RT-qPCR was carried out on a Lightcycler 480^®^ Instrument II (LC480, Roche) using the LightCycler^®^ RNA Master SYBR Green I kit (Roche, Basel, Switzerland). qPCR reactions (20 µL) contained the primers (Table S2) of the target genes at a final concentration of 0.3 µM, 3.25 mM of Mn(OAc)_2_, 1X LightCycler^®^ RNA Master SYBR Green I buffer (Roche, Mannheim, Germany), and two µL of RNA samples. RNA samples were prepared by adjusting at the same concentration and further diluting 1:10 for the phage genes and 1:100 for the 16S rRNA gene of *Salmonella*, which served as the reference gene. RT-qPCR conditions were the same as detailed previously for qPCR but with an initial RT step at 61 °C for 20 min prior to the qPCR reaction. The quantification of mRNA transcripts for target genes was performed using the E-method, which considers the efficiency of the target genes as well as that of the reference gene (16S rRNA) [76] and time 0 as the calibrator. Three independent infection experiments were carried out, and each RNA sample was studied twice in each RT-qPCR experiment. The statistical analysis of the expression of the different genes tested was performed by comparing the expression of genes at each time point between cells that carried the *ibfA* gene and those that did not.

### 4.12. Transmission Electron Microscopy (TEM) of Infected Salmonella Cells

The visualization of the formation of viral capsids inside cells was carried out using TEM. Bacterial cultures were prepared as explained above and infected with UAB_Phi20 at MOI of 1 pfu/cfu. Samples of 10 mL were taken from the cultures at 0, 20, 30, 40, 60 and 80 min after phage adsorption and centrifuged at 12,857× *g* for 10 min. The pellets were suspended in 2 mL fixative solution [2% paraformaldehyde, 2.5% glutaraldehyde in phosphate buffer (0.1 M NaH_2_PO_4_, pH 7.4)] and incubated for at least 2 h at 4 °C. After fixation, the samples were washed four times with phosphate buffer for 10 min. Post-fixation was performed using 1% OsO_4_ overnight at 4 °C. Then, the samples were washed four times with water for 5 min, and 1% uranyl acetate was added to the samples and kept overnight at 4 °C. Finally, the washing step was repeated as previously described. Dehydration was carried out using a series of acetone solutions at increasing concentrations of 50%, 70%, 90%, 96%, and 100%. The samples were then embedded in Epon-815 Resin (Electron Microscopy Sciences, Hatfield, PA, USA) using a series of resin–acetone mixtures at ratios 1:3, 2:2, 3:1 and 4:0, respectively, at room temperature for 2 h each, and finally left overnight with resin. The following day, another change of resin was carried out, and the samples were left for 2 h at room temperature. Polymerization was performed at 70 °C for 48 h. Subsequently, 70 nm sections were obtained using a Reichert-Jung Ultracut E ultramicrotome (C. Reichert Optische Werke AG, Vienna, Austria) and placed on PELCO^®^ copper-coated 200-mesh grids (Ted Pella, Inc., Redding, CA, USA). The stained sections were examined in a Hitachi H-7000 transmission electron microscope at 100 kV at instrumental magnifications of 0.5 or 1 μm (Servei de Microscòpia i Difracció de Raig X, UAB, Barcelona, Spain).

### 4.13. Bioinformatic Studies

Several bioinformatics analyses were carried out to characterize the plasmid pUA1135 genome. A functional category enrichment of plasmid pUA1135 was performed using eggNOG-mapper v2 [77]. PADLOC [35] and DefenseFinder [78,79] were used to determine the presence of known defense systems in plasmid pUA1135. To assess the presence of unknown mechanisms of phage interference, we used tBLASTN against the NCBI plasmid database (PLSDB) to identify thirty homologous plasmids, prioritizing query coverage and an e-value of 1 × 10^−5^. Plasmid sequences were aligned using Mauve [80] and the variable region of each plasmid was identified as the sequence found between *repA* and *parA* genes. To quantify the variability in this region of pUA1135 plasmid, we used hmmscan against the COG database [81,82] to assign clusters of orthologous groups (COG) identifiers to each protein-coding gene in all plasmid sequences (both variable and non-variable regions). Pairwise comparisons between all plasmids were performed, tallying for each plasmid pair the number of shared COGs in both variable and non-variable regions. The variability in each region was then assessed as the ratio between the sum of shared COGs and total sum of COGs for both plasmids in that region.

To infer the phylogeny of IbfA, a BLASTP search was conducted using the IbfA protein sequence against the clustered nr database of bacterial (taxid:2) and archaeal (taxid:2157) protein sequences. Results were filtered to include only hits with >85% query coverage, with an e-value of 1 × 10^−5^, and only those from complete chromosomal or plasmid sequences were kept. Alignments were performed using ClustalX [83] and T-Coffee [84] with default parameters and integrated with T-Coffee. Phylogenetic inference was performed using iqtree [85] with default parameters and annotation of the resulting tree was performed with iTOL [86].

Promoter prediction for *ibfA* and its transcriptional unit was carried out using BPROM [37] and SAPHHIRE.CNN [87] using default parameters. The study of the protein functional domains of IbfA was performed using HHpred [88] against the PDB, PFAM and COG databases and the *E. coli* proteome, with otherwise default parameters. Furthermore, a sequence-based search against AlphaFold AFDB [41,42] was used to find similar proteins with an experimentally obtained function or structure.

### 4.14. Quantification and Statistical Analysis

Differences between means were assessed using the *t*-test with Prism 8.0.2 (GraphPad, La Jolla, CA, USA). Data are depicted as the mean and standard deviation, or as the geometric mean and standard deviation of the mean in the case of gene expression studies. Differences were considered as statistically significant when *p* < 0.05. The parameters and *p*-values are reported in the relevant figure legends.

## Figures and Tables

**Table 1:** EOP values of the UAB_Phi20 bacteriophage for deletion mutants of the IC5 strain.

Strain	EOP ^2^
ATCC14028 Rif^R^	1
IC5 ^1^	0
IC5 Δ *ibfA*	0.94 ± 0.13
IC5 Δ *pUA1135_00003*, *ibfA*, *ydeA*, *ydfA*	0.65 ± 0.07

^1^ This strain contains the pUA1135 plasmid. ^2^ Calculated with respect to the parental strain ATCC14028 Rif^R^. Standard deviation was obtained from three independent replicates.

**Table 2:** EOP values of the UAB_Phi20 bacteriophage for complementation of deletion mutants of the IC5 strain.

Strain	EOP ^1^
IC5 Δ *ibfA*/pBAD33	1
IC5 Δ *ibfA*/pBAD33::*ibfA*	0.05 ± 0.00
IC5 Δ *pUA1135_00003*, *ibfA*, *ydeA*, *ydfA*/pBAD33::*pUA1135_00003*, *ibfA*, *ydeA*, *ydfA*	0.01 ± 0.00

^1^ Calculated with respect to the strain IC5 Δ *ibfA*/pBAD33. Standard deviation was obtained from three independent replicates.

**Table 3:** Mean of virions per cell section and percentage of cell sections with virions in the presence and absence of the *ibfA* gene.

	*ibfA* Gene	40 min	60 min	80 min
Mean of virions/cell section ± SD	−	31 ± 3.6	27 ± 3.7	28 ± 4.3
	+	16 ± 3.7	7 ± 3.5	7 ± 2.9
Percentage (%) of cell sections with virions vs. total of recorded sections	−	32.9	88.8	8.5
	+	2.3	3.6	7.5

The values represent the mean and the standard deviation of 16 fields of 70 nm sections. SD, standard deviation.

## Data Availability

The assembled UAB_Phi20 bacteriophage genome and pUA1135 plasmid sequences were deposited at NCBI, with the following accession numbers: NC_031019 and MW590592, respectively, and are publicly available as of the date of publication. IC5 bacterial genome has been deposited in the SRA archive under the Bioproject: PRJNA746119.

## Supplementary Materials

The following supporting information can be downloaded at: https://www.mdpi.com/article/10.3390/ijms26104918/s1. References [25,64,71,72] are cited in the Supplementary Materials.

## Abbreviations

The following abbreviations are used in this manuscript:

TEM	Transmission electron microscopy
TOPRIM	Topoisomerase-primase
RM	Restriction-modification
PgI	Phage growth limitation
BREX	Bacteriophage exclusion
DISARM	Defense island associated with RM
pAgos	Prokaryotic argonautes
RADAR	Restriction by an adenosine deaminase acting on RNA
CRISPR-Cas	Clustered regularly interspaced short palindromic repeats and associated proteins
pVips	Prokaryotic viperins
Abi	Abortive infection
TA	Toxin-antitoxin
Rif	Rifampicin
dsDNA	Double-stranded DNA
PLSDB	NCBI plasmid database
COG	Clusters of orthologous groups
EOP	Efficiency of plating
Sar	small antisense RNA
Hma	Helicase, methylase, ATPase
ECOI	Efficiency of center of infection
PES	Polyether sulfone
COI	Center of infection
KCN	Potassium cyanide
qPCR	Quantitative real-time PCR
