# Supplementary material for: Deciphering the Causes of IbfA-Mediated Abortive Infection in the P22-like Phage UAB_Phi20
Source: Int J Mol Sci. 2025 May 20;26(10):4918. doi: 10.3390/ijms26104918 (PMC12111858; doi:10.3390/ijms26104918)
Supplement: Supplementary file 1 [file ijms-26-04918-s001.zip › ijms-3615317-supplementary.pdf]

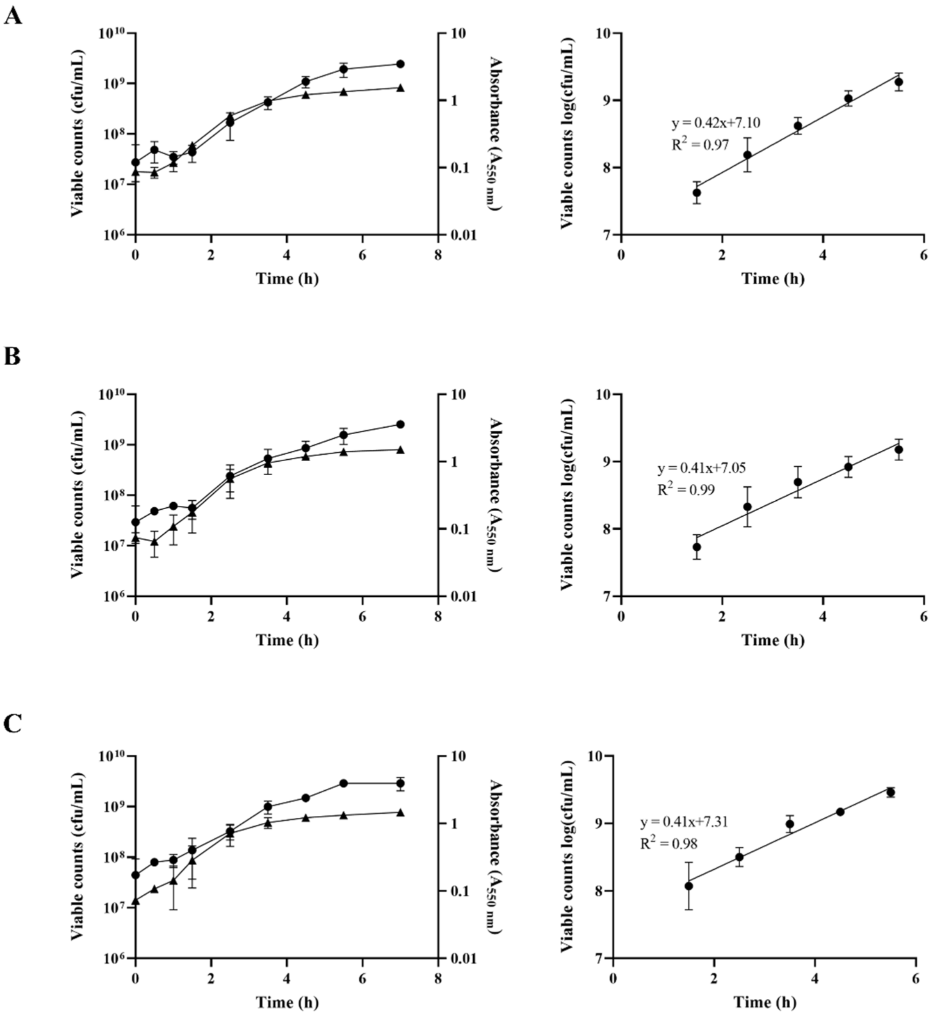

Figure S1. Bacterial growth (logarithmic and linear kinetics) in the absence and presence of *ibfA*. (**A**) Growth kinetics of ATCC14028 Rif^R/pBAD33 strain. (**B**) Growth kinetics of ATCC14028 Rif^R/pBAD33::*ibfA* strain. (**C**) Growth kinetics of ATCC14028 Rif^R. (●), cfu/mL; (▲), absorbance ($A_{550\ nm}$). All experiments were done in duplicate, and the error bars are visualized in the graphs.

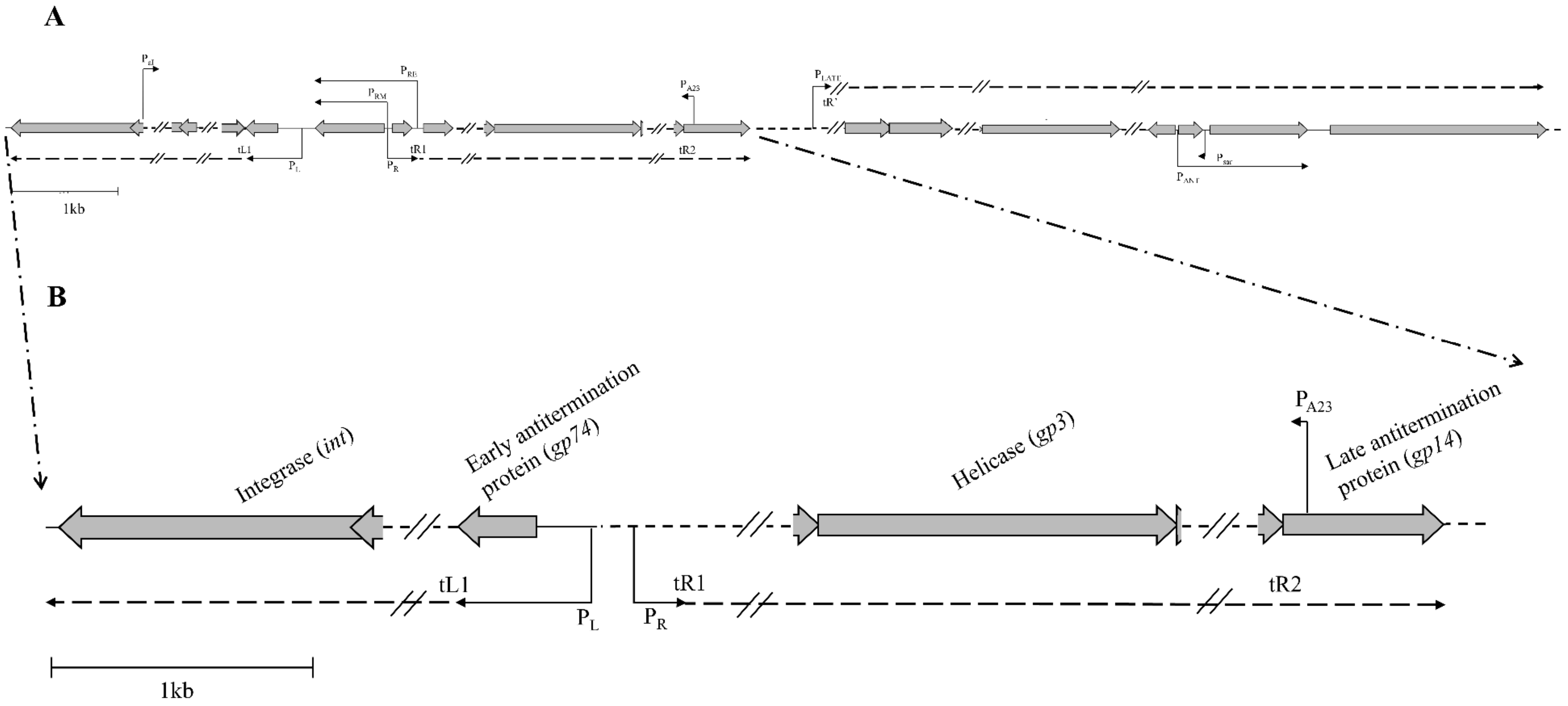

Figure S2. (**A**) Schematic representation of the genome of UAB_Phi20 phage. (**B**) Enlargement of the early genes controlled by P_L and P_R, whose transcription was studied through RT-qPCR.

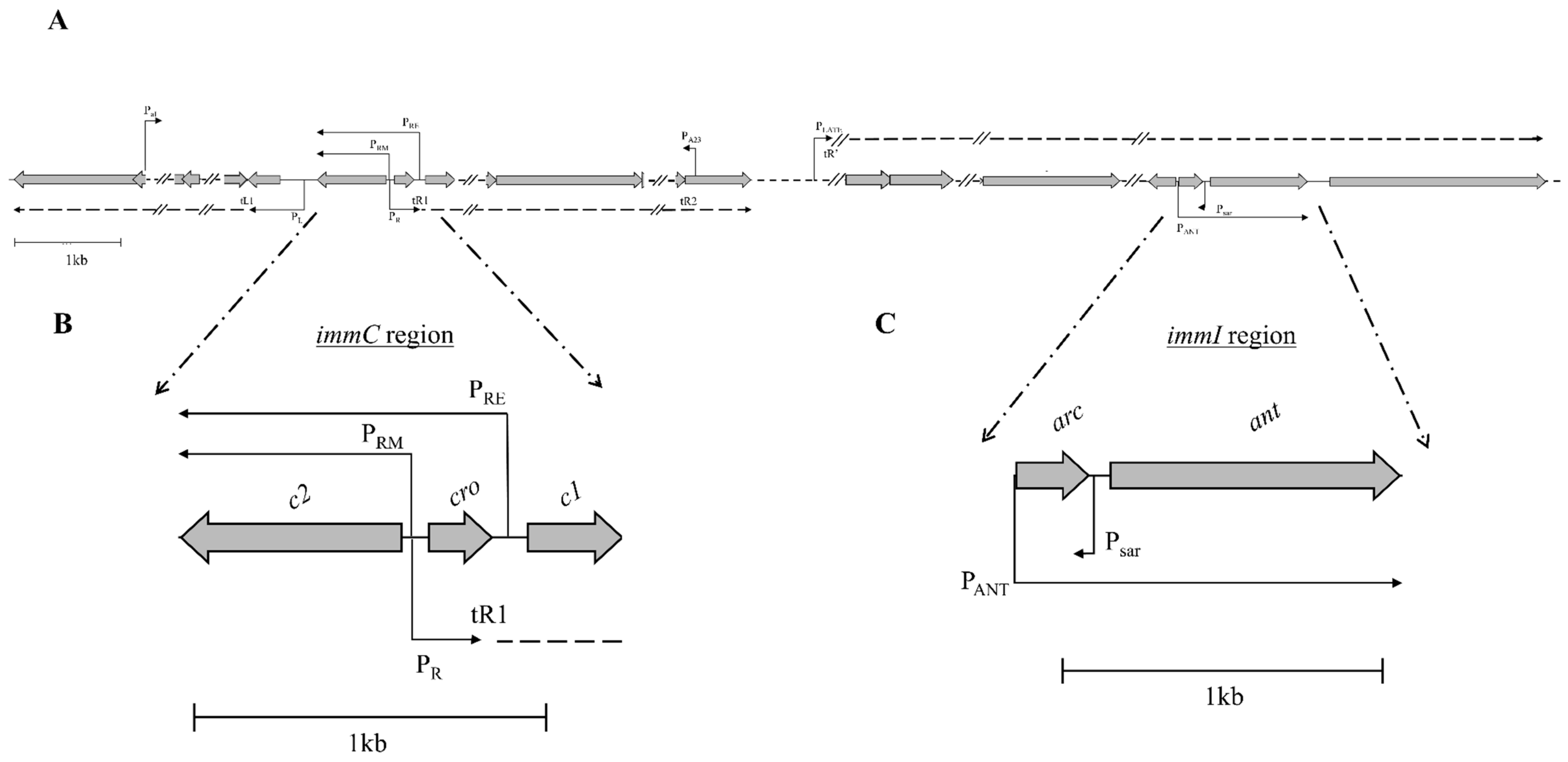

Figure S3. (**A**) Schematic representation of the genome of UAB_Phi20 phage. Enlargement of the *immC* (**B**) and of the *immI* (**C**) regions, whose transcription was studied through RT-qPCR.

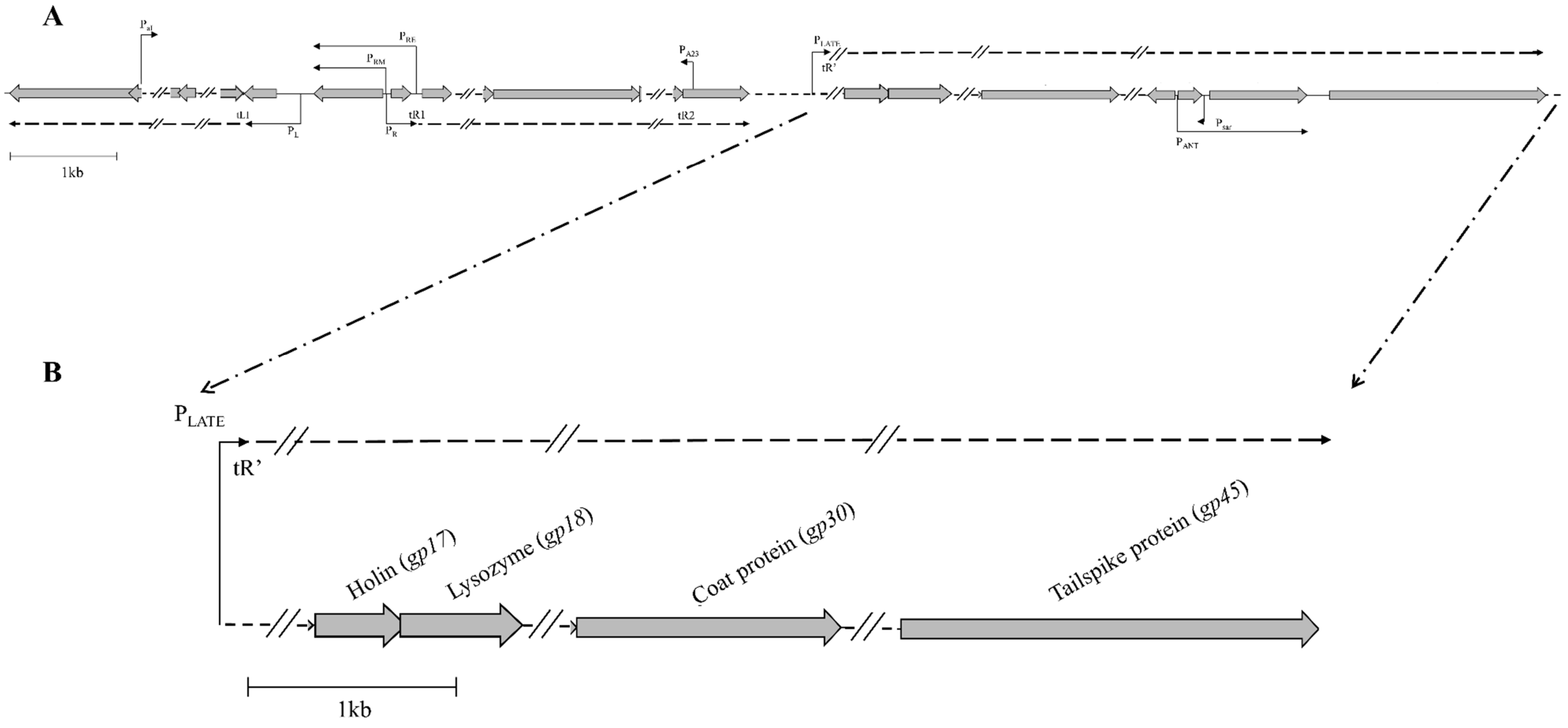

Figure S4. (**A**) Schematic representation of the genome of UAB_Phi20 phage. (**B**) Enlargement of the late genes controlled by P_{LATE}, whose transcription was studied through RT-qPCR.

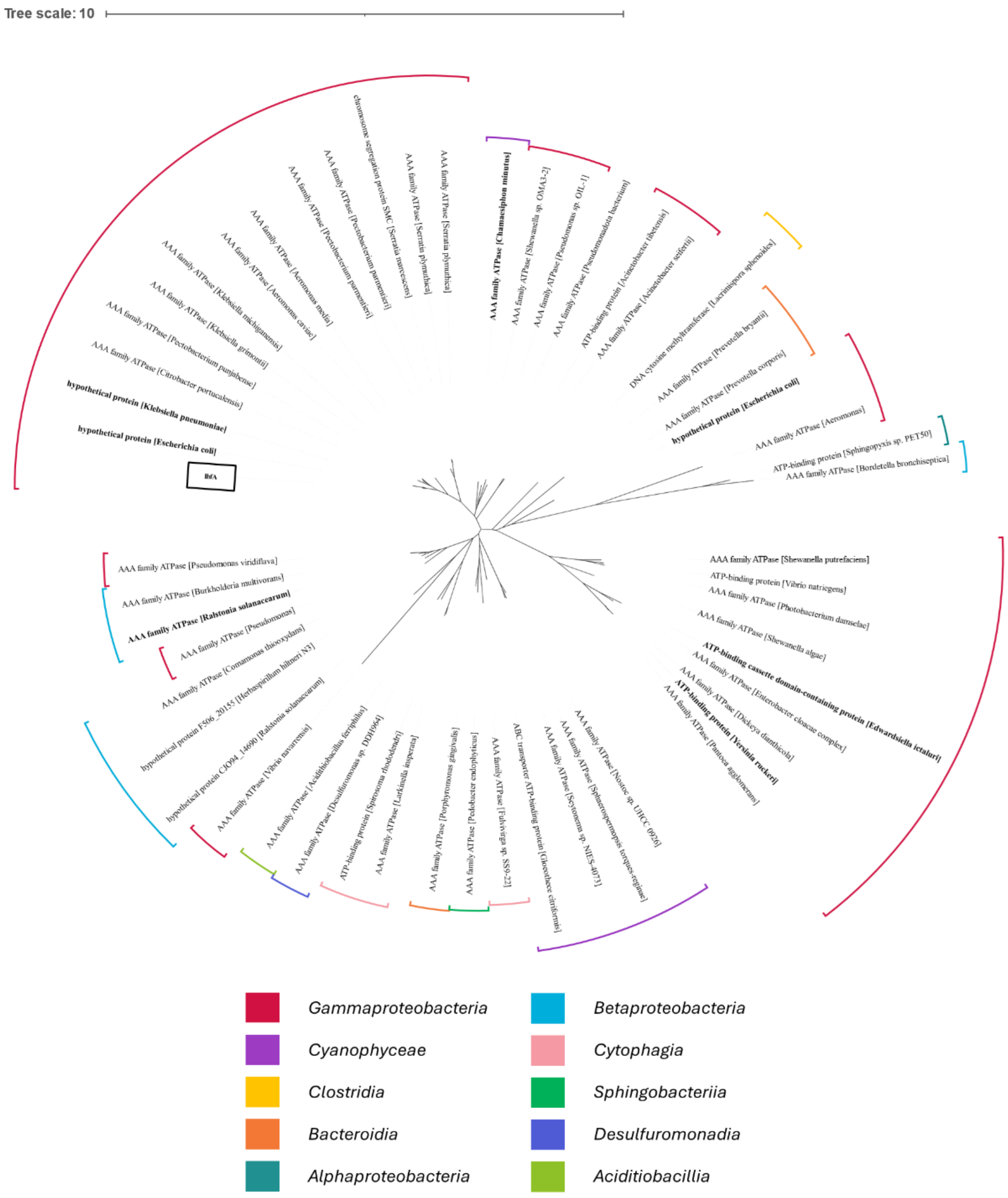

Figure S5. Phylogenetic tree of IbfA homologs in bacterial and archaeal complete plasmids (highlighted in bold) or chromosomes. IbfA protein is marked in a black square. Fifty-five different proteins were included in the tree. The tree scale indicates branch length.

Table S1. List of COGs from the variable region of homologs of the pUA1135 plasmid.

COG	COG annotation	Number of total instances of each COG found at least once in the analyzed plasmids
COG3316	Transposase (or an inactivated derivative)	44
COG4974	Site-specific recombinase XerD	22
COG3109	sRNA-binding protein	15
COG1192	Cellulose biosynthesis protein BcsQ	14
COG4637	Predicted ATPase	14
COG4271	Predicted nucleotide-binding protein containing TIR -like domain	12
COG2367	Beta-lactamase class A	10
COG2814	Predicted arabinose efflux permease, MFS family	7
COG0030	16S rRNA A1518 and A1519 N6-dimethyltransferase RsmA/KsgA/DIM1 (may also have DNA glycosylase/AP lyase activity)	6
COG3039	Transposase and inactivated derivatives, IS5 family	6
COG0294	Dihydropteroate synthase	5
COG0459	Chaperonin GroEL (HSP60 family)	5
COG1662	Transposase and inactivated derivatives, IS1 family	5
COG0262	Dihydrofolate reductase	4
COG0270	Site-specific DNA-cytosine methylase	4
COG0645	Predicted kinase	4
COG0840	Methyl-accepting chemotaxis protein	4
COG1309	DNA-binding transcriptional regulator, AcrR family	4
COG2076	Multidrug transporter EmrE and related cation transporters	4
COG2746	Aminoglycoside N3'-acetyltransferase	4
COG3385	IS4 transposase	4
COG4644	Transposase and inactivated derivatives, TnpA family	4
COG0399	dTDP-4-amino-4,6-dideoxygalactose transaminase	3
COG1961	Site-specific DNA recombinase related to the DNA invertase Pin	3
COG3173	Predicted kinase aminoglycoside phosphotransferase (APT) family	3
COG3843	Type IV secretory pathway, VirD2 components (relaxase)	3
COG0480	Translation elongation factor EF-G, a GTPase	2
COG0583	DNA-binding transcriptional regulator, LysR family	2
COG1357	Uncharacterized protein YjbI, contains pentapeptide repeats	2
COG1670	Protein N-acetyltransferase, RimJ/RimL family	2
COG2801	Transposase InsO and inactivated derivatives	2
COG3231	Aminoglycoside phosphotransferase	2
COG3547	Transposase	2
COG3570	Streptomycin 6-kinase	2
COG4584	Transposase	2
COG0110	Acetyltransferase (isoleucine patch superfamily)	1
COG0491	Glyoxylase or a related metal-dependent hydrolase, beta-lactamase superfamily II	1
COG0640	DNA-binding transcriptional regulator, ArsR family	1
COG0768	Cell division protein FtsI/penicillin-binding protein 2	1

Table S1 (continued)

COG	COG annotation	Number of total instances of each COG found at least once in the analyzed plasmids
COG0786	Na+/glutamate symporter	1
COG1476	DNA-binding transcriptional regulator, XRE-family HTH domain	1
COG1484	DNA replication protein DnaC	1
COG1695	DNA-binding transcriptional regulator, PadR family	1
COG1708	Predicted nucleotidyltransferase	1
COG1715	Restriction endonuclease Mrr	1
COG2059	Chromate transport protein ChrA	1
COG2329	Heme-degrading monooxygenase HmoA and related ABM domain proteins	1
COG4747	Uncharacterized conserved protein, contains tandem ACT domains	1
COG4875	Uncharacterized protein	1
COG4977	Transcriptional regulator GlxA family, contains an amidase domain and an AraC-type DNA-binding HTH domain	1

Table S2. Strains and plasmids used in this study.

Strain	Relevant characteristics	Source or reference
ATCC14028 Rif^R	*S.* Typhimurium; Rif^R	This research group
DH5*α*	*E. coli supE4 ΔlacU169* (φ80 *ΔlacZ ΔM15*) *hsdR17, recA1, endA1, gyrA96, thi-1, relA1*	Clontech
IC5	ATCC14028 Rif^R variant; Amp^R	[25]
ATCC14028 Rif^R pBAD33	*S.* Typhimurium with pBAD33 vector; Rif^R, Cm^R	This work
ATCC14028 Rif^R pBAD33::*ibfA*	*S.* Typhimurium with pBAD33 vector containing *ibfA* gene cloned with its own promoter; Rif^R, Cm^R	This work
**Plasmid**	**Relevant characteristics**	**Source or reference**
pKD4	Amp^R Km^R	[71]
pKOBEG	Vector containing the *λ* Red recombinase system, Cm^R, thermosensitive	Generous gift of Prof. I. Lasa; [72]
pBAD33	Low copy number vector containing the arabinose inducible pBAD promoter, Cm^R	[64]
pBAD33::*ibfA*	pBAD33 derivative containing the *ibfA* gene cloned under the control of its own promoter, Cm^R	This work
pBAD33::*pUA1135_00003, ibfA, ydeA, ydfA*	pBAD33 derivative containing the *ibfA*-containing transcriptional unit cloned under the control of its own promoter, Cm^R	

Amp, ampicillin; Cm, chloramphenicol; Km, kanamycin; Rif, rifampicin; R, resistant.

Table S3. Primers used in this work.

Name	Sequence (5′-3′)	Application
ibfA_km_p1.2 [1]	TTTCTGAATTACATGGTGAATTCCCCCAACATAATGCTAGGATTTAAAAATG*gtgtaggctggagctgcttc*	PCR of Km gene from pKD4 plasmid with homology regions of the *ibfA* gene
ibfA_km_p2 [1]	TATTTGCTCATTCGGGATGGTTGGATATTTTAAGTTATATCTACTGTATC*atgggaattagccatggtcc*	
ibfA_ext_fw2	GAGGTTCGCGTACAGATAA	PCR and sequencing to check the Km insertion into the *ibfA* gene.
ibfA_ext_rv2	CCATCTTTACCGTCTCTGAA	
UT_km_p1 [1]	CTTCACAATGAATATCATTGTGTTGGTAACCTGCTGTTATCGTGATGAAA*gtgtaggctggagctgcttc*	PCR of Km gene from pKD4 plasmid with homology regions of the *ibfA*-containing transcriptional unit.
UT_km_p2 [1]	ACATGATATGATTTTTACCTGTCAGAACATTATCAGATGGAGCGTGCAGG*atgggaattagccatggtcc*	
UT_ext_fw	GTACTTGCCACAGCAGGT	PCR and sequencing to check the Km insertion into the *ibfA*-containing transcriptional unit.
UT_ext_rv	TGTGCTAACGGCATGTATTAC	
00003_ibfA_fw	ATAATGATGGGTGTTTTCTTTTCT	Primers used in RT-PCR to determine the formation of a transcriptional unit.
00003_ibfA_rv	AGCCCGTAGTTTCTGATTT	
ibfA_ydeA_fw	CTTGACCAGTAATAGTTTTT	
ibfA_ydeA_rv	CGCGTACAGATAAATGAAA	
ydeA_ydfA_fw	GGCATCCTCCAGACGAC	
ydeA_ydfA_rv	TGAATGCCATAAAGAGACAG	
ibfA_P1_pBAD33_fw [2]	*agcgaattcgagctcggtac*TTAAGTTATATCTACTGTATCATTCTCAGGTAATCGTTC	PCR of *ibfA* gene from pUA1135 plasmid used to clone this gene into pBAD33 vector under the control of its own promoter.
ibfA_P1_pBAD33_rv [2]	*tctagaggatccccgggtac*GAACACGCCTGTGCACGG	
pUA1135-Big4_pBAD33_f [2]	*gctcggtacccggggatcctctagagtcgacctg*GAAAGGAATACTTCACAATGAATATCATTG	PCR of *ibfA*-containing transcriptional unit from pUA1135 plasmid used to clone into pBAD33 vector under the control of its own promoter.
pUA1135-Big4_pBAD33_r [2]	*cttctctcatccgccaaaacagccaagcttgcatgcctg*CCTATTATCTCCGTGTTAGCCG	
pBAD_F	ATGCCATAGCATTTTTATCC	PCR and sequencing to check the introduction of *ibfA* or the *ibfA*-containing transcriptional unit in pBAD33 vector
pBAD_R	CCTGATACAGATTAAATCAG	
sec_ibfA_P1_1	GCAGCTTCAAACGCATTCA	
sec_ibfA_P1_2	CACGAATAAGTGTACTGGCA	
sec_ibfA_P1_3	CGGCAGTGACTTCATTGT	
sec_ibfA_P1_4	CTCGAACATCAGCTTTTCCA	

[1] P1 and P2 sequences, homologs to the pKD4 plasmid, are represented in lower case italics; [2] Homology sequences to pBAD33 are represented in lower case italics.

Table S3. (continued).

Name	Sequence (5′-3′)	Application
gyrBupRT	GTTTTTGCGACAGAGCGTTGAC	Quantification of ATCC14028 Rif^R *gyrB* through qPCR
gyrBdwRT	ACATGGTATTCGAGGTGGTAGA	
16SRNAupSa	CACATGCAAGTCGAACGGTAAC	Quantification of ATCC14028 Rif^R 16S rRNA through RT-qPCR
16SRNAdwSa	AGTGTGGCTGGTCATCCTCTCA	
ibfA_P1_RT_fw	TCACCGGCACAGAATCAC	Quantification of ATCC14028 Rif^R pBAD33::*ibfA ibfA* gene through RT-qPCR
ibfA_P1_RT_rv	TCCACCCAATCCGTCCAGT	
Phi20_gp03_RT_fw	CCGGCGCAGGAATGATGTC	Quantification of UAB_Phi20 helicase through qPCR and RT-qPCR
Phi20_gp03_RT_rv	CGTTCCGCTTTTGGTTTCTC	
Phi20_gp17_RT_fw	CATGATGGCGGCAAAGGAAC	Quantification of UAB_Phi20 holin expression through RT-qPCR
Phi20_gp17_RT_rv	TCTGTGCCGATGTAGCCGATAAAC	
Phi20_gp18_RT_fw	AGCAGGGGGATACCAACCAT	Quantification of UAB_Phi20 lysozyme expression through RT-qPCR
Phi20_gp18_RT_rv	CGGGACGCGAACAAGACTAC	
Phi20_gp30_RT_fw2	GAAACCATCTCCGCAATCAC	Quantification of UAB_Phi20 coat protein expression through RT-qPCR
Phi20_gp30_RT_rv2	GCAGTCTCGTCTCGCAAGTC	
Phi20_gp45_RT_fw	CAGCGGTCGTTGCCACTTT	Quantification of UAB_Phi20 tailspike protein expression through RT-qPCR
Phi20_gp45_RT_rv	GACCGCTAGCCCGATGAACT	
Phi20_gp14_RT_fw	GGGAATGGCGCAATCACAAG	Quantification of UAB_Phi20 late transcription antitermination protein expression through RT-qPCR
Phi20_gp14_RT_rv	CGCGGCACTACGGCAATAAT	
Phi20_gp74_RT_fw	CCATAGAGCGCGACACCATC	Quantification of UAB_Phi20 early transcription antitermination protein expression through RT-qPCR
Phi20_gp74_RT_rv	CCTCCTTCAACTTCCTTCTG	
Phi20_gp43_RT_fw	CCACGGTGGACCTGTATTGT	Quantification of UAB_Phi20 *arc* expression through RT-qPCR
Phi20_gp43_RT_rv	CAATGCGCCCTTCCTTCTTA	
Phi20_gp44_RT_fw	ACCGCTGGCCGTAGAGTA	Quantification of UAB_Phi20 antirepressor *ant* expression through RT-qPCR
Phi20_gp44_RT_rv	GAATTCATGGCCGTAGTCGT	
Phi20_sar_RT_fw	GTGTTAACTGCTTCTAAAATTGCTA	Quantification of UAB_Phi20 *sar* expression through RT-qPCR
Phi20_sar_RT_rv	GAAGCCCCAACTGCGGTAAC	
Phi20_gp77_RT_fw	ATTAGCGATGCAGCGGTCTC	Quantification of UAB_Phi20 *cro* expression through RT-qPCR
Phi20_gp77_RT_rv	CTTGGCGATAAGCGTTTTCTTG	
Phi20_gp51_RT_fw	CCGGCTTAGGCTTGAATACC	Quantification of UAB_Phi20 integrase expression through RT-qPCR
Phi20_gp51_RT_rv	AGAAGGCGCATAAGAAGTCG	

[1] P1 and P2 sequences, homologs to the pKD4 plasmid, are represented in lower case italics; [2] Homology sequences to pBAD33 are represented in lower case italics.

Table S3. (continued).

Name	Sequence (5′-3′)	Application
Phi20_C1_RT_fw	ACGCAATTACCAGCAGCATC	Quantification of UAB_Phi20 *c1* expression through RT-qPCR
Phi20_C1_RT_rv	ATCCTCGACACCCCACTC	
Phi20_C2_RT_fw	GAATGTGGCCTCGTTTTCAC	Quantification of UAB_Phi20 *c2* expression through RT-qPCR
Phi20_C2_RT_rv	TAAGCGCAGGGCAATGGAT	

[1] P1 and P2 sequences, homologs to the pKD4 plasmid, are represented in lower case italics; [2] Homology sequences to pBAD33 are represented in lower case italics.
